# Evaluation of Skin Hardness as a Physiological Sign of Human Thermal Status

**DOI:** 10.1038/s41598-018-30206-1

**Published:** 2018-08-13

**Authors:** Sunghyun Yoon, Jai Kyoung Sim, Noeul Park, Young-Ho Cho

**Affiliations:** 1 Department of Bio and Brain Engineering, Korea Advanced Institute of Science and Technology (KAIST) 291 Daehak-ro, Yuseong-gu, Daejeon 34141 Republic of Korea; 2 Present Address: 711th Human Performance Wing, Air Force Research Laboratory, Wright-Patterson AFB, Ohio 45433 United States

## Abstract

This is the first ever proposal to use skin hardness as a physiological sign by which to estimate human thermal status and to verify its effectiveness and independence in relation to the two conventional signs: skin temperature and skin conductance. We propose a novel TSV model adding skin hardness to the conventional TSV model for better estimation of human thermal status with higher accuracy and lower error. We survey individual thermal sensation from 30 subjects under four different thermal conditions (normal, warm, hot, and cold); while measuring skin hardness along with the two conventional physiological signs. The novel model for estimation of thermal status from all three signs increases *R*^2^ by 17.4% and decreases error by 23.5%, compared to the conventional model using two signs. The novel TSV model has potential for applications to human-machine interaction systems for better estimation of human thermal status.

## Introduction

Currently, the monitoring of human thermal status is in demand for systems involving human-machine interaction, such as cognitive air conditioning systems^1–11^ which control the surrounding air temperature based upon human thermal status (HTS). Conventional HTS monitoring is performed on the basis of a survey of individual thermal sensation, called a ‘Thermal Sensation Vote’ (TSV)^12,13^. The conventional TSV models estimate individual thermal sensation with one^12–20^ or two^21–23^ physiological signs (i.e. skin temperature^12–19^ and/or skin conductance^20^). However, the conventional thermal status estimation models that use a single physiological sign show a *Coefficient of Determination* (*R*^2^: 0 ≤ *R*^2^ ≤ 1) of less than 0.6^14,21^. This is not good enough for accurate HTS estimation using TSV. The TSV model using two physiological signs (skin temperature and skin conductance^24^) shows enhanced accuracy, but still shows low estimation accuracy (*R*^2^ < 0.7).

In this paper, we propose to add a novel physiological sign (skin hardness) to the conventional TSV model with two physiological signs. We assert that this should not only improve its accuracy but should also to reduce the error rate of the TSV-based estimation of HTS. The proposal in the present study is based on the increase and decrease in skin hardness due to the contraction and relaxation (respectively) of the arrector pili muscles. The arrector pili muscles are connected to the hair follicles in human skin, and their contraction pulls the skin and increases skin density, thereby increasing skin hardness. The arrector pili muscle are known to contract or relax depending on human thermal status^25^; therefore, the skin has different hardness depending on the human individual thermal status (HTS).

We intend to verify that the skin hardness is independent of the conventional physiological signs of skin temperature and skin conductance. In addition, we suggest a novel TSV model, in which skin hardness is added to the two conventional signs. We evaluate and compare the accuracy and error of the novel TSV model with those of conventional TSV estimation models. To our knowledge, this is the first ever attempt to estimate the human thermal status based on skin hardness.

## Results

### Experimental Purposes and Methods

The experiment was designed to achieve three goals: (1) Verify the independence of skin hardness in relation to the two conventional physiological signs of skin temperature and skin conductance, (2) Verify the effectiveness of skin hardness, compared to the two conventional signs, for estimating human thermal status and (3) Evaluate the novel TSV model including skin hardness compared to the conventional models of skin temperature and skin conductance.

We surveyed individual TSV (Table S1) from 30 subjects (Table S2) under four different thermal conditions (i.e. reference room, warm room, hot room, cold room; Table S3), while measuring skin hardness along with skin temperature and skin conductance. The subjects were moved to three rooms (warm, hot and cold) passing through the reference room in a way that exposed them to seven consecutive stages of measurement (Fig. S1). The three physiological signs (skin hardness, skin temperature, and skin conductance) were measured on the wrist and arm of each subject.

All physiological signs and TSV were normalized by subject and stage. The performance of five TSV models (TSV models 1–5) was evaluated in relation to the physiological data. The five TSV models for data from wrist and arm were regressed respecting skin hardness, skin temperature, skin conductance, both skin temperature and skin conductance, and all three signs (hardness, temperature and conductance). The five wrist TSV models were denoted by W1, W2, W3, W4 and W5, while the five arm TSV models were denoted as A1, A2, A3, A4 and A5. The two sets of five TSV models each were compared on the basis of five analytical values: (1) Regression Coefficient, (2) Coefficient of Determination (*R*^2^), (3) Adjusted Coefficient of Determination (*R*_*Adj*_^2^), (4) Variance Inflation Factor (VIF), and (5) Root Mean Square Error (RMSE) from the 7-fold leave-out cross-validation test.

### Experimental Results

The regression coefficients of the two sets of five TSV models have p-value under 0.0001, thereby demonstrating their statistical relevance. Table 1 shows the analytic values (i.e. *R*^2^, *R*_*Adj*_^2^, and RMSE) of the wrist and the arm TSV models, respectively. Table 2 includes the VIFs (4 and 5) of the wrist and arm TSV models, respectively.

**Table 1:** Statistical wrist and arm models for the TSV (Thermal Sensation Vote) estimation based on the skin hardness, skin temperature, and/or skin conductance.

Wrist TSV model ID	Derived TSV model equation (X_i_^*^ = normalized value of i)	Coefficient of determination (R^2^) < Adjusted coefficient of determination (R_Adj_^2^)>	Root mean square error (RMSE) [%]	Arm TSV model ID	Derived TSV model equation (X_i_^*^: normalized value of i)	Coefficient of determination (R^2^) < Adjusted coefficient of determination (R_Adj_^2^)>	Root mean square error (RMSE) [%]
W1 (hardness)	−0.760*X*_*hardness*_ + 0.820	0.6302 <0.6279>	19 ± 1.6	A1 (hardness)	−0.607*X*_*hardness*_ + 0.809	0.4427 < 0.4393>	24 ± 2.5
W2 (temperature)	0.690*X*_*temperature*_ + 0.153	0.5414 <0.5386>	21 ± 1.8	A2 (temperature)	0.690*X*_*temperature*_ + 0.191	0.5590 < 0.5563>	21 ± 2.9
W3 (conductance)	0.666*X*_*conductance*_ + 0.348	0.6176 <0.6152>	19 ± 1.9	A3 (conductance)	0.632*X*_*conductance*_ + 0.353	0.5109 < 0.5080>	22 ± 2.7
W4 (temperature and conductance)	0.373*X*_*temperature*_ + 0.453*X*_*conductance*_ + 0.201	0.7127 <0.7092>	17 ± 2.3	A4 (temperature and conductance)	0.464*X*_*temperature*_ + 0.377*X*_*conductance*_ + 0.194	0.6816 < 0.6777>	18 ± 2.6
W5 (hardness, temperature, and conductance)	−0.438*X*_*hardness*_ + 0.170*X*_*temperature*_ + 0.368*X*_*conductance*_ + 0.501	0.8369 <0.8338>	13 ± 1.3	A5 (hardness, temperature, and conductance)	−0.295*X*_*hardness*_ + 0.357*X*_*temperature*_ + 0.312*X*_*conductance*_ + 0.399	0.7577 < 0.7532>	16 ± 1.5

^*^ $${{\rm{X}}}_{{\rm{i}}}=\frac{{\rm{x}}-{{\rm{x}}}_{{\rm{\min }}}}{{{\rm{x}}}_{{\rm{\max }}}-{{\rm{x}}}_{{\rm{\min }}}}$$

x: measurement value of i.

x_min_: minimum value of i in the subject, x_max_: maximum value of i in the subject.

**Table 2:** Variance inflation factors at the wrist and the arm.

Location	TSV model ID	Parameter	Variance inflation factor
Wrist	W4	Skin temperature	1.66553
		Skin conductance	1.66553
	W5	Skin hardness	1.68409
		Skin temperature	2.04620
		Skin conductance	1.74670
Arm	A4	Skin temperature	1.48630
		Skin conductance	1.48630
	A5	Skin hardness	1.37551
		Skin temperature	1.66350
		Skin conductance	1.55677

## Discussion

Skin hardness is affected by skin diseases such as psoriasis vulgaris^26^ and scleroderma^27^. However, in case of healthy skin, skin hardness is affected by the properties of both elastic fibres and collagen, and arrector pili muscle contraction and extension. The properties of the elastic fibres and collagen^28^ change with the aging state of an individual^29,30^, and have a long-term effect on skin hardness. The contraction/extension of the arrector pili muscles has a short-term effect on skin hardness. In the present study, the short-term effect is predominant for skin hardness because the human subjects in the experiment changed their thermal status within 15 minutes in the seven measurement stages (Fig. S1). This occurred within two hours (overall time of the experiment).

Skin hardness was found to be independent from conventional physiological signs for estimation of HTS. If any VIF among the physiological signs exceed ‘5’, the signs used in the TSV model are inter-correlated^31^. For Model 5 (includes skin hardness, temperature and conductance), the VIF was in the range 1.68–2.04 and 1.38–1.66 at the wrist and arm, respectively. This indicates that the skin hardness is a physiological sign independent from skin temperature and skin conductance. The skin hardness independence is also explained by the fact that the contraction or expansion of ‘arrector pili muscles’ results in more dominant influence on skin hardness, than do changes due to the temperature or conductivity of skin.

The physiological signs estimating the human thermal status include peripheral blood flow, skin temperature, sweat rate, and skin conductance. Peripheral blood flow^32^ and sweat rate^33^ is highly correlated with skin temperature and skin conductance, respectively. In addition, skin temperature^14^ and skin conductance^21^ are the most common conventional physiological signs for the human thermal status estimation. Therefore, we evaluated the effectiveness of skin hardness as a physiological sign for HTS estimation, compared with skin temperature and skin conductance. At the wrist and the arm (Table 1), similar values of *R*^2^ and *R*_*Adj*_^2^ were obtained for all three signs, as were similar absolute values of the coefficient of the derived TSV model equation. This indicates that the effectiveness of skin hardness is equivalent to that of skin temperature and skin conductance.

**Figure 1:**
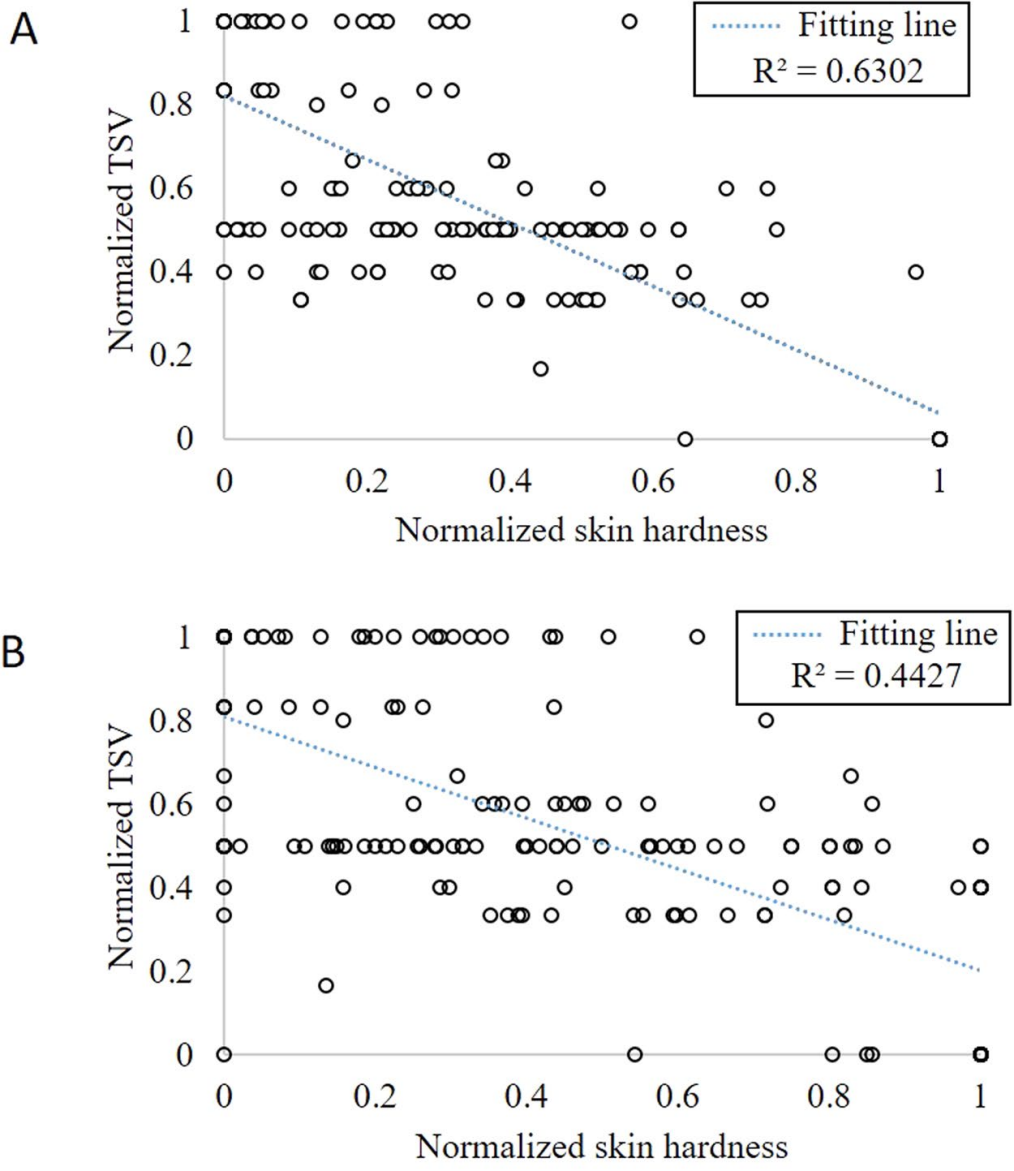
Thermal sensation vote (TSV) and skin hardness correlation, measured from 30 subjects at (**A**) the wrist, and (**B**) the arm.

The skin hardness measured at the wrist serves as a better physiological sign than that at the arm for HTS estimation (Fig. 1). At the wrist, the RMSE of the skin hardness (W1) is lower (by 9.5%) than that of the skin temperature (W2), and the *R*^2^ of W1 is higher (by 8.9%) than that of W2. At the wrist, the RMSE of the skin hardness (W1) is similar with that of the skin conductance (W3), and the *R*^2^ of W1 is 2.0% higher than that of W3.

At the arm, the RMSE of the skin hardness (A1) is higher (by 14.3%) than that of the skin temperature (A2), and the *R*^2^ of A1 is lower (by 20.8%) than that of A2. At the arm, the RMSE of the skin hardness (A1) is higher (by 9.1%) than that of the skin conductance (A3) and the *R*^2^ of A1 is lower (by 13.4%) than that of A3. The experimental results show that the skin hardness at the wrist is more effective and consistent for using TSV to estimate HTS than at the arm.

**Figure 2:**
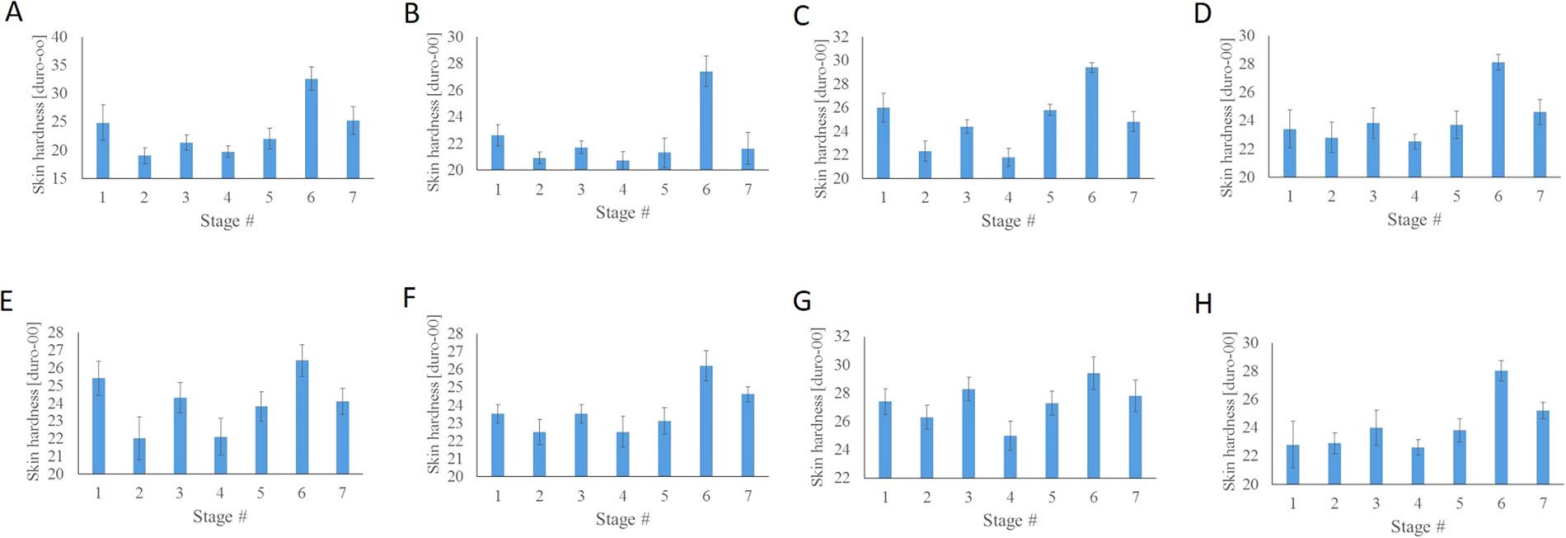
The skin hardness measured at the wrists of 8 different subjects (**A**~**H**) at the 7 stages of Fig. S1.

Figure 2 shows the skin hardness measured at the wrists of eight different typical subjects in the seven measurement stages shown in Fig. S1. At the wrist, the average of the maximum change of individual skin hardness under the four thermal conditions, was 6.32 + 2.31 duro00; thus, the coefficient of variation (CV) was 36.6%. At the arm, the average of the maximum change of individual skin hardness under the same conditions was 4.72 + 2.34 duro00; thus, the CV was 49.6%. The CV at the wrist was lower (by 26.2%) than that at the arm, showing that the skin hardness at the wrist was more consistent than at the arm, for use with the TSV.

We suggest a novel TSV model (for W5) including all three physiological signs (skin hardness, skin temperature and skin conductance), for estimation of human thermal status. This model includes skin hardness as well as the conventional HTS estimation model W4, which uses only skin temperature and skin conductance. W5 enabled more accurate estimation of HTS, reducing the error by 23.5% and increasing *R*^2^ by 17.4%, compared to W4. The *R*^2^ of the novel TSV model was 0.8338, which is higher than that from previous HTS estimation research. In most of the previous research on estimation of HTS using TSV, regression values in the range 0.6–0.7 were reported. For example, L. Lan *et al*.^14^ reported the regression value of 0.74 when using skin temperature; whereas C. P. Chen *et al*.^21^ reported regression values of 0.70 and 0.65 for skin temperature and skin conductance, respectively.

In addition, we performed HTS estimation using the novel TSV model in relation to 1) gender and 2) skinfold thicknesses. The TSV estimation results depending on gender (Table S4) do not show large differences between male and female subjects. Moreover, the data from all seven female subjects and seven randomly chosen male subjects were used to remove the bias from unequal numbers of male and female subjects. The coefficient of determination of the female and male groups were 0.8934 and 0.8529, respectively. The coefficient of determination for the females was higher (by 4.05%) than that of the males. As a result of the additional TSV estimation relative to skinfold thickness (Table S4), the novel TSV model was found to fit well with subjects whose skins were thick. The subjects were categorized into groups with thick skin or thin skin based on the average skinfold thickness (3.329 mm) of the 22 subjects. The coefficient of determination of the thick skin group and thin skin group was 0.8715 and 0.7925, respectively. The novel TSV model was found to fit well with both thick and thin skinfold thickness groups. The coefficient of determination of the thick skin group was higher (by 10.0%) than that of the thin skin group. The effect of the skin fold thickness on the novel TSV model with the male subjects was additionally estimated to remove the bias from gender. The male subjects were categorized into thick skinfold thickness group (n = 8) and thin skinfold thickness group (n = 8) based on the average skinfold thickness (3.186 mm). As a result of the additional estimation, the coefficients of determination (R^2^) of the thick skin group and thin skin group were 0.8602 and 0.8149, respectively, thereby the novel TSV model is found to fit well with thick skinfold thickness group by 5.6%, showing both groups had high R^2^ for human thermal status estimation.

In summary, we proposed the use of skin hardness as a new physiological sign for the estimation of human thermal status and presented a novel HTS estimation model that included skin hardness as well as the two conventional signs, skin temperature and skin conductance. We focused on the skin hardness, which reflects ‘arrector pili muscle’ contraction and expansion in response to HTS. The conventional TSV model (based only on skin temperature and skin conductance) showed low accuracy for HTS estimation. We proposed a novel TSV model in which skin hardness was added to the conventional TSV model for better HTS estimation with higher accuracy and lower error. The TSV model analysis proved that human skin hardness is independent from the other signs. It also showed that skin hardness is an effective physiological sign that improves HTS estimation, compared to the use of only skin temperature and skin conductance. The novel TSV model increased *R*^2^ by 17.4% and decreased error by 23.5% (compared to the conventional model with two factors). The novel TSV model with higher accuracy and lower error has potential for application to systems involving human-machine interaction for better estimation of human thermal status.

## Materials and Methods

### Subjects and measurement conditions

Thirty subjects, twenty-three men and seven women, participated in the experiment. The average subject age, height and weight (Table S2) were 24.2 + 4.3 year, 171.4 ± 7.5 cm and 64.0 ± 9.3 kg, respectively. All subjects in the present research were Koreans 20–30 years old, with BMI in the range 18.5–24.9. All subjects had no disease, including skin trouble, and all was recruited using the IRB (KH2011-18) guidelines.

The present study considered two personal factors (cloth insulation and metabolic heat condition) based on ISO7730^34^. All the subjects wore same clothing: light socks, underwear (bras and panties, half-slip) and Shirt (T-shirt), where the clothing insulation score was 0.047 m^2^ °C/W (0.3 clo). All subjects reclined on the floor for 30 min before starting the experiment. Therefore, the metabolic heat score was 46 W/m^2^ (0.8 met).

The experiment consisted of seven stages of measurement, each composed of two steps: a resting step (10 min) and a measuring step (5 min) with the subjects lying in the supine position. At each step, multiple measures of the skin hardness (5x), skin temperature (3x) and skin conductance (3x) of the subjects were performed, followed by the TSV survey (Table S1).

### Methods and sites for physiological sign measurement

Figure S2 shows the physiological signs measured on the skin of arm or wrist. Skin hardness was measured using the Durometer 00 (GS-754G, Teclock, Japan). This device is a type adaptable for the hardness measurement of soft materials like human skin. The durometer has a 1.19 mm radius hemisphere indenter and a spring with adjustable loading (203–1111 mN). The Durometer 00 was mounted in a specially designed jig (Fig. S2) composed of a polystyrene body and two stainless steel legs. The jig had two functions: 1) Maintaining contact force with a mass of 400 g between the durometer and the subject skin (based on the standard durometer guideline ASTM D2240) and 2) Supporting the durometer upright to the skin of the subjects for the stable measurement. Figure S3 shows the specific spots used for measurement of the selected signs. The wrist skin hardness was measured at the spot located on the centre line of the dorsal wrist, 3 cm from the line A–A′ passing the radial styloid process. The arm skin hardness was measured at the spot located on the centre line of the brachial arm, 7 cm from the crook of the arm (line B–B′).

The skin temperature was measured using calibrated thermocouples (TC-K, *Innpitron*, South Korea) connected to a digital multimeter (Fluke 289, *Fluke*, USA). For each subject, identical thermocouples were used. The thermocouples were attached to the subject’s skin using a semi-permeable patch (Opsite Flexifix, S*mith & Nephew*, UK). At the wrist and arm, the skin temperature was measured at a spot on the dorsal centre line, 5 cm from the spot used for the skin hardness measurement.

Skin conductance was measured at the wrist and arm using a couple of skin attachable electrodes (Kendall 450 foam electrodes, *Covidien*, USA) 1 cm apart from the right and left edges of the skin temperature electrode.

Skinfold thickness was measured using a caliper based on the skinfold thickness measurement method^35^. The skinfold thickness was measured at the same spot as the skin hardness measurement at the wrist and the arm. The skinfold thickness was measured three times for each subject and the average of the three measurement values was used in the statistical analysis.

### Statistical analysis of physiological signs

Measurements of the physiological signs and TSV were normalized for each subject and each stage to minimize the individual deviation of each subject. The normalized physiological signs were regressed on the normalized TSV for statistical analysis. The regression analysis (SAS 9.4, *SAS Institute Inc*., USA) was utilized to analyse the performance of the TSV models based on the normalized signs.

### Human experiment

KAIST Institutional Review Board (IRB) approved the present human experiments. All the physiological measurements performed on human subjects were carried out with informed consent under the guidelines and regulations of the KAIST IRB, ID number KH2011–18.

## Electronic supplementary material

Supplementary information
