# Supplementary material for: Evaluation of Skin Hardness as a Physiological Sign of Human Thermal Status
Source: Sci Rep. 2018 Aug 13;8:12027. doi: 10.1038/s41598-018-30206-1 (PMC6089900; doi:10.1038/s41598-018-30206-1)
Supplement: Supplementary file 1 — Supplementary information [file 41598_2018_30206_MOESM1_ESM.pdf]

Evaluation of Skin Hardness as a Physiological Sign of Human Thermal Status

Sunghyun Yoon[+], Jai Kyoung Sim[+], Noeul Park, and Young-Ho Cho[*]

Department of Bio and Brain Engineering,

Korea Advanced Institute of Science and Technology (KAIST)

291 Daehak-ro, Yuseong-gu, Daejeon 34141, Republic of Korea

Tel.: +82-42-350-8691 / Fax: +82-42-350-8690 / E-mail: nanosys@kaist.ac.kr

[+] Authors who contributed equally

[*] Corresponding author

Keywords: Skin hardness, human thermal sensation, physiological sign, thermal comfort status

Table S1 Thermal sensation vote

Stage	Test room ID	Subject's thermal sensation level						
		-3 (Cold)	-2 (Cool)	-1 (Slightly Cool)	0 (Neutral)	1 (Slightly Warm)	2 (Warm)	3 (Hot)
1	Room 1							
2	Room 2							
3	Room 1							
4	Room 3							
5	Room 1							
6	Room 4							
7	Room 1							

Table S2 Subject information

	Male (n_m=23)	Female (n_f=7)	Average ($n=n_m+n_f$=30)
Age [year]	25.3 ± 4.2	20.6 ± 2.0	24.2 ± 4.3
Height [cm]	173.6 ± 6.3	164.1 ± 6.8	171.4 ± 7.5
Weight [kg]	67.1 ± 7.9	53.7 ± 5.1	64.0 ± 9.3
BMI* [kg/m^2]	22.2 ± 2.0	19.9 ± 0.7	21.7 ± 2.1

***Body Mass Index(BMI) = Weight / Height^2**

Table S3 Test room conditions

Conditions	Test room ID (Thermal states)			
	Room 1 (reference)	Room 2 (warm)	Room 3 (hot)	Room 4 (cold)
Air temperature [°C]	28.1 ± 1.2	39.7 ± 4.1	47.7 ± 2.4	-2.1 ± 1.6
Relative humidity [%]	45.2 ± 10.5	38.8 ± 4.9	29.1 ± 4.2	50.1± 8.9
Mean radiant temperature [°C]	25.4 ± 1.9	35.3 ± 2.6	41.9 ± 1.6	15.7 ± 3.1
Wind velocity [m/s]	< 0.5	< 0.5	< 0.5	< 0.5

Table S4 Coefficient of Determination of the Novel TSV model based on gender

	Male (n = 7*)	Female (n=7)
Coefficient of Determiation (R^2)	0.8529	0.8934
Adjusted coefficient of determination (R_{Adj}^2)	0.8431	0.8848

*Randomly chosen subjects

Table S5 Coefficient of Determination of the Novel TSV model based on skinfold thickness at the wrist

	Group of thick skinfold thickness (> 3.329 mm*)	Group of thin skinfold thickness (≤ 3.329 mm*)
Coefficient of determination (R^2)	0.8715	0.7925
Adjusted coefficient of determination (R_{Adj}^2)	0.8667	0.7829

*Subject's average skin thickness

Table S6 Subjects information of Table S5

	Group of thick skinfold thickness		Group of thin skinfold thickness	
	Male	Female	Male	Female
Subject number	8	4	8	2
Average skinfold thickness (mm)	3.615	4.102	2.758	2.866

Table S7 Coefficient of Determination of the Novel TSV model based on skinfold thickness with male subjects at the wrist

	Group (n = 8) of thick skinfold thickness (> 3.186 mm*)	Group (n = 8) of thick skinfold thickness (≤ 3.186 mm*)
Coefficient of determination (R^2)	0.8602	0.8149
Adjusted coefficient of determination (R_{Adj}^2)>	0.8521	0.8040

*Subject's average skin thickness

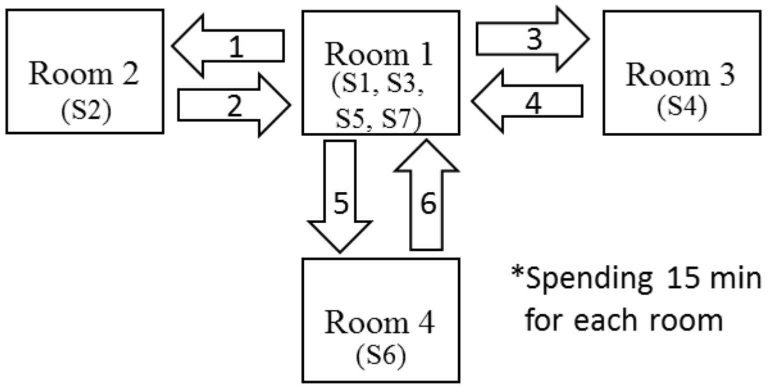

Figure S1 Experiment protocol, composed of the 7 measurement stages of S1~S7 following the paths from 1 to 6 among 4 test rooms.

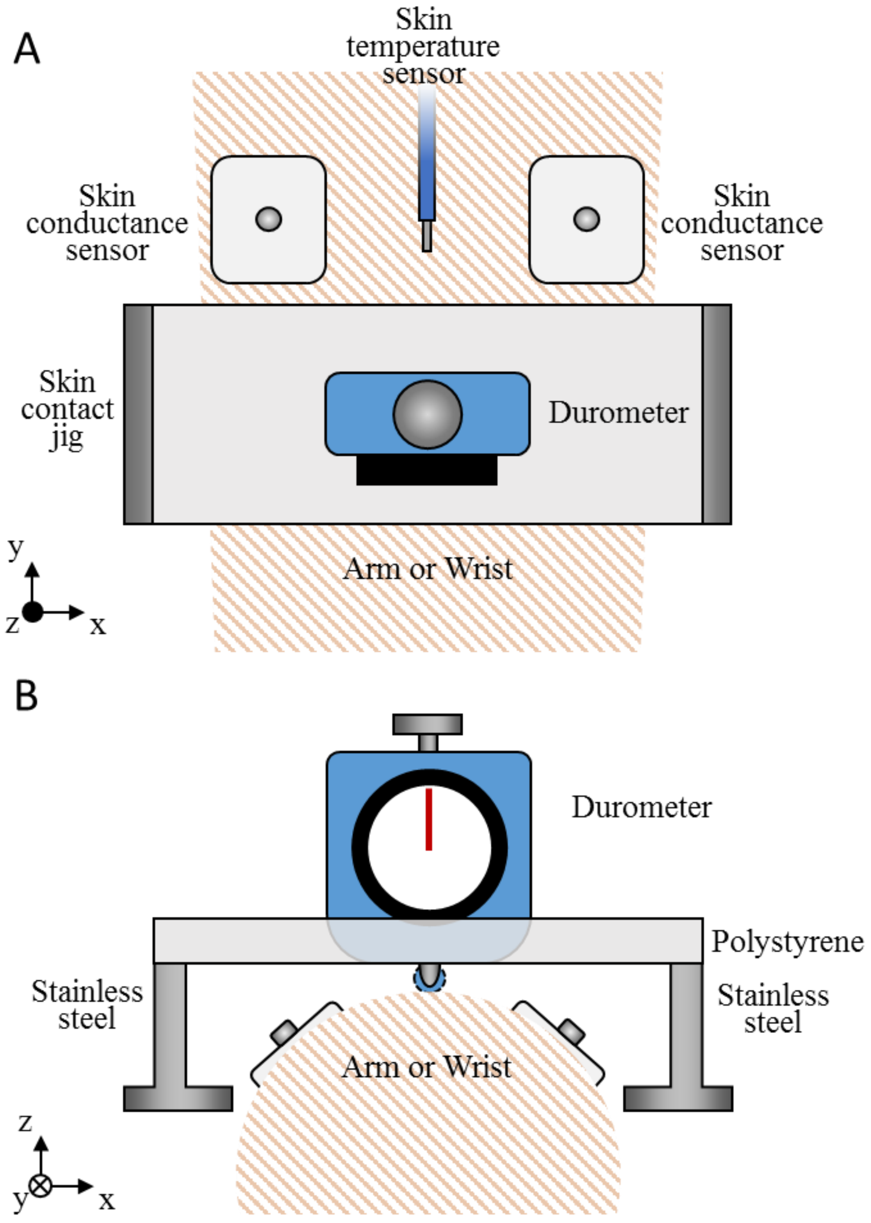

Figure S2 Physiological signs (skin hardness, skin temperature and skin conductance) measurement on the human skin of arm or wrist: (A) top view, (B) side view.

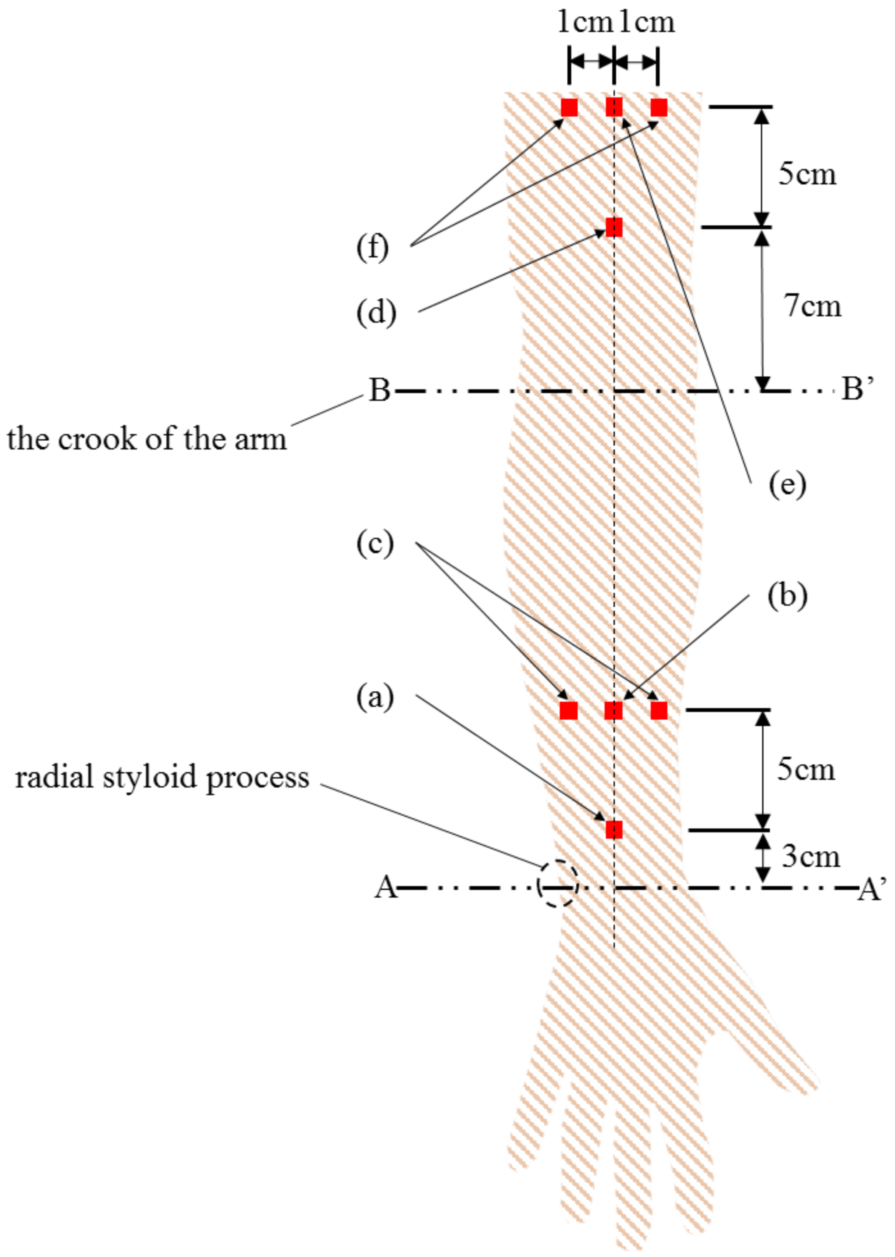

Figure S3 The physiological sign measurement spots: (a) the wrist skin hardness; (b) the wrist skin temperature; (c) the wrist skin conductance; (d) the arm skin hardness; (e) the arm skin temperature; (f) the arm skin conductance.
